# Recent Developments and Applications of Microbial Levan, A Versatile Polysaccharide-Based Biopolymer

**DOI:** 10.3390/molecules28145407

**Published:** 2023-07-14

**Authors:** Marta Domżał-Kędzia, Monika Ostrowska, Agnieszka Lewińska, Marcin Łukaszewicz

**Affiliations:** 1 Faculty of Biotechnology, University of Wroclaw, Joliot Curie 14a, 50-383 Wroclaw, Poland; 2 Research and Development Department InventionBio S.A., Jakóba Hechlińskiego 4, 85-825 Bydgoszcz, Poland; monika.ostrowska@inventionbio.pl; 3 Faculty of Chemistry, University of Wroclaw, Joliot Curie 14, 50-383 Wroclaw, Poland; 4 OnlyBio Life S.A., Jakóba Hechlińskiego 6, 85-825 Bydgoszcz, Poland

**Keywords:** levan, biopolymers, polysaccharide, *Bacillus subtilis*, safety, food industries, pharmaceutical industries, cosmetics

## Abstract

Polysaccharides are essential components with diverse functions in living organisms and find widespread applications in various industries. They serve as food additives, stabilizers, thickeners, and fat substitutes in the food industry, while also contributing to dietary fiber for improved digestion and gut health. Plant-based polysaccharides are utilized in paper, textiles, wound dressings, biodegradable packaging, and tissue regeneration. Polysaccharides play a crucial role in medicine, pharmacy, and cosmetology, as well as in the production of biofuels and biomaterials. Among microbial biopolymers, microbial levan, a fructose polysaccharide, holds significant promise due to its high productivity and chemical diversity. Levan exhibits a wide range of properties, including film-forming ability, biodegradability, non-toxicity, self-aggregation, encapsulation, controlled release capacity, water retention, immunomodulatory and prebiotic activity, antimicrobial and anticancer activity, as well as high biocompatibility. These exceptional properties position levan as an attractive candidate for nature-based materials in food production, modern cosmetology, medicine, and pharmacy. Advancing the understanding of microbial polymers and reducing production costs is crucial to the future development of these fields. By further exploring the potential of microbial biopolymers, particularly levan, we can unlock new opportunities for sustainable materials and innovative applications that benefit various industries and contribute to advancements in healthcare, environmental conservation, and biotechnology.

## 1. Introduction

The exploration of biopolymers and their applications has gained significant attention in various fields, including food, cosmetics, pharmaceuticals, drug delivery systems, biomedical applications, tissue engineering, and environmental protection [1,2,3,4]. Such research aims to develop multifunctional materials that are safe for human use, reducing reliance on synthetic polymers and mitigating their environmental impact. Among the biopolymers, there are main groups such as polysaccharides, proteins or nucleic acids. Polysaccharides are compounds consisting of simple sugars linked together by glycosidic bonds. Polysaccharides can be divided into two main types based on their composition: homo- and heteropolysaccharides. The first type of polysaccharides is homopolysaccharides, which consist of repeating units of the same monosaccharide [5]. Examples of homopolysaccharides include glycogen, which serves as a glucose storage form in animals and fungi and consists of a large chain of glucose molecules, and cellulose, which is a structural component of plant cell walls. Cellulose consists of long chains of glucose units linked by β-glycosidic bonds [6]. Another example of homopolysaccharides is starch, a storage polysaccharide found in plants, fruits and seeds. It consists of two components, amylose and amylopectin, which are made up of glucose units. An additional example is inulin, composed of fructose units, found in tubers of plants such as dahlia and artichoke, where it acts as a storage polysaccharide. The second type is heteropolysaccharides composed of different types of monosaccharide units. Examples of heteropolysaccharides include hyaluronic acid composed of repeating units of D-glucuronic acid and N-acetyl-glucosamine. Hyaluronic acid is found in connective tissue and plays a role in moisturizing and lubrication. Another example of heteropolysaccharides is heparin, which consists of D-glucuronic acid, L-iduronic acid and N-sulfo-D-glucosamine. It is primarily found in mast cells and blood, where it acts as an anticoagulant. Gamma globulin is a heteropolysaccharide consisting of N-acetyl-hexosamine, D-mannose and D-galactose units. It is found in the blood and plays a role in the immune response. By understanding the distinction between homopolysaccharides and heteropolysaccharides, we can appreciate the diversity and functional roles of polysaccharides in various biological systems.

Polysaccharides have a variety of biological functions and are important components of various biological systems [5]. Thus, polysaccharides can also be divided based on their functional roles in organisms. The two main categories are storage polysaccharides and structural polysaccharides. Storage polysaccharides are primarily involved in storing energy as food supplies. They act as a readily available source of energy when needed. Examples of storage polysaccharides include mainly glycogen and starch [7]. Structural polysaccharides are involved in providing support and structure to cells and tissues. They contribute to the formation of various structural components of organisms. Examples of structural polysaccharides, among others, encompass cell wall components such as cellulose, hemicellulose, and lignin [8].

Polysaccharides are widely used due to their unique properties and abundance. Some notable applications include paper and board production, electrical insulation, organic fertilizer production, pharmaceutical stabilizers, textiles and clothing, and food and personal care products. For example, cellulose is a key ingredient in the production of various paper products, including paper, paperboard and cardboard. Its fibrous structure allows the creation of a strong and flexible material that is widely used in packaging, printing and many other industries [6]. Chitin is used in the production of organic fertilizers. Chitin-based fertilizers are environmentally friendly, non-toxic and biodegradable. They can improve soil health and promote sustainable agriculture by increasing nutrient availability, stimulating microbial activity and increasing crop productivity [9]. Starch plays an important role in many industries, including the beer brewing process. During beer production, starch is converted into fermentable sugars by the enzymatic action of amylases. These sugars are then fermented by yeast to produce alcohol and carbon dioxide [10]. Xylan is used in various industries due to its unique properties and conversion products. It is a valuable source of xylitol, a sugar substitute widely used in the food and pharmaceutical industries. Xylan can be extracted from lignocellulosic biomass, such as agricultural residues and wood waste, as part of a biorefinery process. As soil additives and plant growth enhancers, xylan-based products are used in agriculture and horticulture. Xylan-based materials can improve soil structure, water retention and nutrient availability, leading to increased crop productivity and plant health [11]. In the case of heteropolysaccharides, the chondroitin sulfate commonly used as a dietary supplement in alternative medicine in the treatment of osteoarthritis, and heparin used in the treatment of venous thromboembolism, should be distinguished [12]. Studies utilizing mouse models of cystic fibrosis have demonstrated the potential of hyaluronic acid to alleviate the inflammation commonly associated with this disease [13]. Hyaluronic acid is also widely used in cosmetic and dermatological treatments such as dermal fillers and skincare products due to its ability to retain moisture and improve skin hydration [13]. 

Research by Wickramaarachchi et al. indicates the use of alginate for storing electricity [14]. To enable the transition to clean energy using renewable but intermittent energy sources such as solar and wind power, it is necessary to store energy to guarantee a constant supply of energy. In their study, the researchers investigated the potential of Electrolytic Manganese Dioxide (EMD) composites synthesized from different baths containing alginate as a biopolymer additive for electrochemical supercapacitors. The addition of alginate to the EMD composites had a significant impact on both the physical and electrochemical storage properties of the materials, as compared to the pristine EMD. The cross-linked alginate EMD composite exhibited a remarkable fivefold increase in capacitance, measuring 487 F/g, at a current density of 1 mA/cm^2^ in a three-electrode system with a 2 M NaOH aqueous electrolyte. This improvement demonstrates the enhanced energy storage capabilities of the EMD/Alg composite. To further support their findings, the researchers employed a simulation model of molecular dynamics, which confirmed the influence of alginate on the interactions between ions and polymers in the electrolytic bath. Simulations showed that alginate provides a template for binding Mn^2+^ ions in a relatively ordered manner. This may help the EMD/Alg composite grow under more favorable conditions for energy storage applications [14].

**Figure 1:**
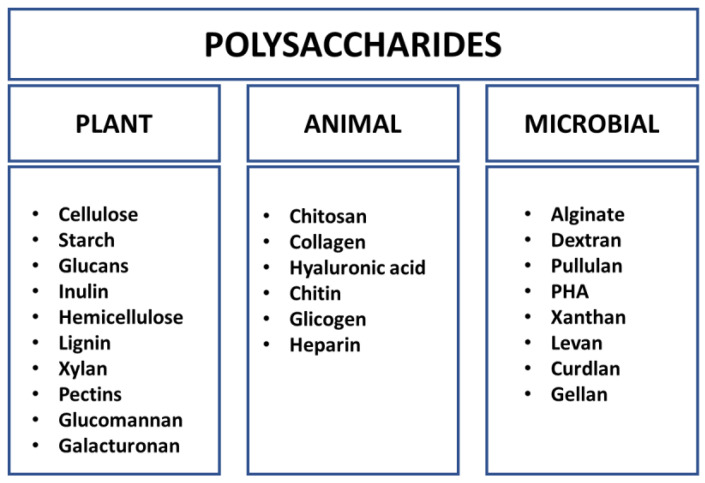
Classification of polysaccharides according to their origin.

Polysaccharides are ubiquitous in plants, animals, and microorganisms (Figure 1). However, with the ongoing depletion and destruction of the natural environment, including the reduction in both the area and diversity of plant and animal species, there is a growing need for alternatives to compounds derived from these sources. As a result, polysaccharides of microbial origin are emerging as a compelling alternative.

Microorganisms have always accompanied man, constituting an inseparable element of existence. The needs emerging along with the development of civilization and the knowledge gained have led not only to the development of methods of combating pathogens but also to the use of their huge potential for the synthesis of useful products, including polysaccharides. The physical and chemical properties of these polysaccharides vary depending on their origin, leading to various classifications: intracellular, structural, exocellular, homopolysaccharides, heteropolysaccharides, branched, rectilinear, anionic, neutral or cationic polysaccharides. Polysaccharides serve as stabilizers, emulsifiers, gelling and coagulating agents, and can be employed to create thin coatings for diverse surfaces. The diverse array of polysaccharides offers immense potential for application across various industries (Table 1). Notably, microbial polysaccharides such as alginate, dextran, pullulan, xanthan and levan [15] have garnered attention due to their ability to utilize by-products from various industrial processes. Moreover, these polysaccharides possess properties that rival or even surpass those of synthetic polymers, particularly in terms of their biological attributes. The broad spectrum of applications stemming from the physical and chemical properties of polysaccharides underscores their significance in industry, underscoring the need for further research to enhance our understanding of their production. This review focuses on introducing levan, highlighting its distinctive characteristics, preparation methods and its potential applications. By delving into the potential of levan, this study aims to contribute to the exploration and utilization of this remarkable polysaccharide. This paper aims to present the achievements in the levan study in recent years.

## 2. Biosynthesis of Levan

**Figure 2:**
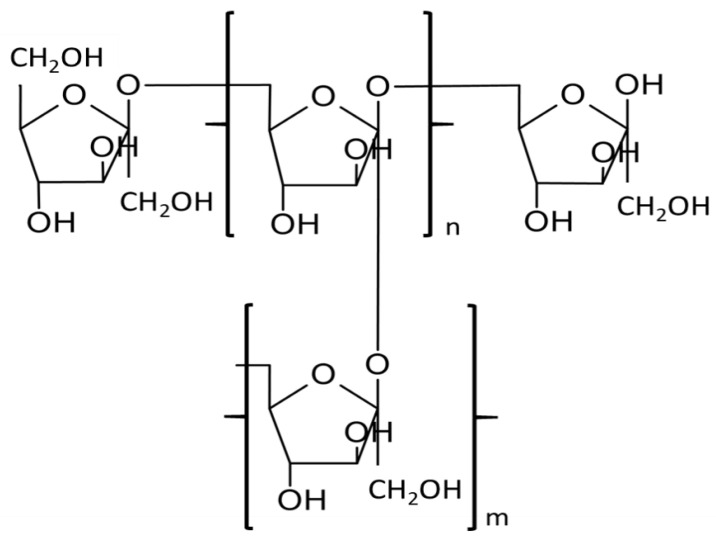
The structural formula of levan.

Levan is a polysaccharide made up of fructose molecules connected by β-2,6-glycosidic bonds in the main chain and β-2,1 in branching (Figure 2). Levan biosynthesis involves the hydrolysis of sucrose into glucose and fructose polymer through the action of the enzyme levansucrase. Levansucrase catalyzes the transfer of fructose units from sucrose to form β-2,6 glycosidic linkages, resulting in the formation of levan. Levan is primarily digested by the enzyme levanase, which breaks down the β-2,6 glycosidic linkages, releasing fructose as the main metabolite. Levan is synthesized from sucrose by various microorganisms, e.g., bacteria *Zymomonas mobilis*, *Erwinia herbicola*, *B. subtilis* or fungi such as *Aspergillus sydowii*, *Aspergillus versicolor*, and it is also found in plants [26,27,28]. Bacterial levans often have molecular weights over 500,000 Da, are commonly branched and form compact nanospheres offering a broad spectrum of applications [26,29].

**Figure 3:**
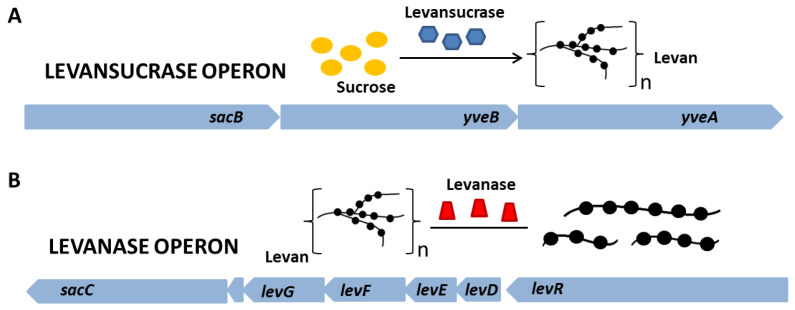
Organization of operons important in the production of levan by *B. subtilis*.

**Figure 4:**
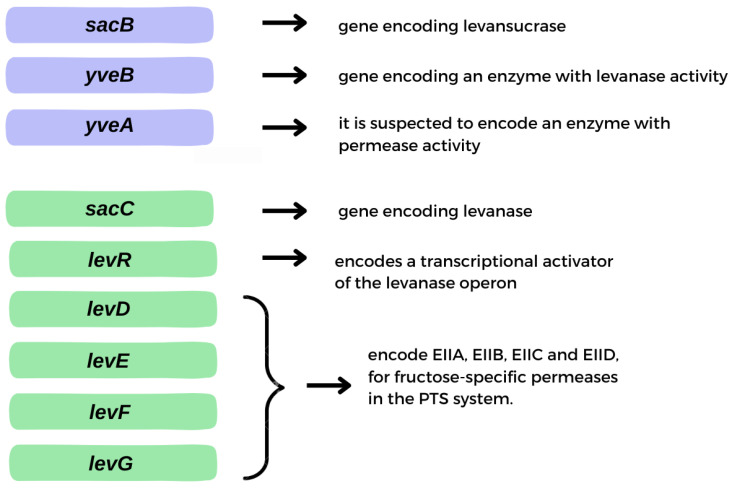
Genes essential in levan synthesis and hydrolysis.

Levan can be produced by microbial fermentation by many microorganisms exhibiting levansucrase activity (EC 2.4.1.10) and can also be synthesized from sucrose by various levansucrase enzymes in raw, purified, recombinant or immobilized form. To date, levan biosynthesis has been reported using levansucrase from many microorganisms, especially strains of *Bacillus*, *Geobacillus*, *Lactobacillus* and *Zymomonas* species. Levansucrase is an enzyme responsible for the hydrolysis of sucrose molecules and the catalysis of transfructosylation reactions using either a sucrose molecule or fructooligosaccharide as an acceptor [30,31]. The enzyme is synthesized within the cytoplasm and subsequently accumulates in the periplasm, where it undergoes final conformational changes before secretion [32]. Secretion of levansucrase occurs via the SecA pathway, and its expression level directly impacts the production of levansucrase, indicating a close relationship between the biosynthesis pathway and the secretory pathway [33]. Genes important in the synthesis and hydrolysis of levan are located in two operons: levansucrase and levanase (Figure 3A,B). The gene encoding levansucrase (*sacB*) is located in a tricistronic operon containing, in addition to the *sacB* gene, the *levB* gene (*yveB*), encoding an enzyme with levanase activity, and the *yveA* gene, whose function has not yet been determined. *yveA* is suspected to encode an enzyme with permease activity, due to its similarity to other already-known proteins (Figure 4) [34,35]. The enzyme encoded by the *levB* gene has (according to the literature) endo-levanase activity, causing the hydrolysis of high molecular weight (HMW) levan molecules to oligosaccharides with a low degree of polymerization (1–10 kDa), down to levanotriosis, levanobiosis and to a small extent fructose molecules [36,37]. It hydrolyzes β-2,6-glycosidic bonds in the polymer, without interfering with β-2,1 bonds in branching [37]. Levanase encoded by *levB* remains bound to the cell membrane [36].

It should be mentioned that levansucrase, in addition to the synthesis activity, also has levanase activity, disrupting β-2,6 bonds, stopping the hydrolysis after reaching β-2,1 bonds in branching [34]. This action targets LMW particles of levan, with a mass no higher than 10^6^ Da [38]. The simultaneous action of enzymes encoded by the *sacB* and *levB* genes on levan results in an increased release of fructose molecules. This indicates their joint regulation—the action of *levB* as endo-levanase stimulates the activity of *sacB* exo-levanase. Working together, the two proteins mimic the *sacC* activity relative to levan [39].

The second important operon for levan is the levanase operon. Levanase is encoded by the *sacC* gene and is located in an operon consisting of five genes—*sacC*, *levD*, *levE*, *levF* and *levG*. It has the activity of exo-levanase, degrading levan molecules via hydrolysis of β-2,6 bonds in molecules that have at least three interconnected fructose molecules [34,40]. Its expression is induced by the presence of fructose [41]. The *levR* gene encodes the transcriptional activator of the levanase operon. Genes *levD*, *levE*, *levF* and *levG* encode EIIA, EIIB, EIIC and EIID, respectively, fructose-specific permeases in the PTS system (Figure 4). In addition, *levE* controls *levR* activity [41,42,43,44,45]. *B. subtilis* synthesizes levan with bimodal molecular weight distribution—HMW and low molecular weight (LMW) polymer fractions are formed [46]. Their synthesis occurs simultaneously, and the mechanism of this reaction is not yet well understood [47]. One of the hypotheses for the formation of two polymer fractions in one reaction system is the synthesis of HMW by enlarging the previously synthesized LMW levan [46]. Euzenat et al. considered this possibility, also noting the possible low affinity of levansucrase for LMW levan, leading to its accumulation. The slow transfer of several fructosyl residues to the LMW levan molecules eventually lengthens LMW to reach HMW levan [48]. The second hypothesis assumes that the synthesis of HMW and LMW occurs through two different elongation processes. The mechanism of elongation is partly determined by the concentration of levansucrase [46]. High sucrose concentrations favor the synthesis of LMW levan (up to ∼70 kDa), while HMW synthesis may occur at low enzyme concentrations [46]. It is worth noting that levan produced by different organisms has different average molecular weights with varied ranges, as well as the degree of branching, which affects its properties and possible applications [49].

A novel levansucrase-producing levan from *Brenneria goodwinii* was also characterized. Genomic DNA *B. goodwinii*, which was recently completed and deposited in the GenBank database, contains the putative gene encoding levansucrase, CPR14579.1. A full-length gene encoding levansucrase was commercially synthesized and cloned into the expression vector pET-22b(+). In this study, recombinant levansucrase *B. goodwinii* was purified and characterized as a levan polysaccharide. The high concentration of sucrose significantly promoted the polymerization reaction to produce the biopolymer. The purified recombinant enzyme produced 185 g/L levan from 50% (*w*/*v*) sucrose at pH 6.0 and 35 °C for 12 h. The molecular weight of the produced levan polysaccharide reached 1.3 × 10^8^ Da, which was much higher than the ones produced by many reported levansucrases [50].

Research by Gao et al. describes the synthesis of levan by *Clostridium acetobutylicum*. The gene encoding levansucrase (*Ca-sacB*) consists of 1287 bp and encodes 428 amino acid residues. Interestingly, *Ca-sacB* was found to have a high product specificity, and fructooligosaccharide was not identified in the product, indicating that *Ca-sacB* may be valuable in industrial levan production. In addition, *Ca-sacB* is the first characterized levansucrase isolated from anaerobic bacteria, which should be valuable for the discovery of new enzymatic resources and advancing understanding of the catalytic mechanisms of this enzyme [51].

Synthesis of levan by *Leuconostoc mesenteroides* has also been demonstrated. Researchers from the Ishida team cloned the gene encoding levansucrase and confirmed that the product of the recombinant enzyme was levan, which is a type of secreted exopolysaccharide that contributes to IgA induction in mice [52].

Kirtle et al. points out that the halophilic bacteria *Halomonas smyrnensis* can synthesize this polysaccharide. The gene encoding levansucrase (*LSC*) was cloned into a vector with 6xHis Tag at the C terminus and then expressed in *Escherichia coli*. The purified recombinant levansucrase produced levan and a wide range of fructooligosaccharides from sucrose, but only in the presence of high salt concentrations (>1.5 M NaCl). Due to its unique biochemical properties, the enzyme from this bacterial strain is important for future research and potential industrial applications in a world facing drought and dwindling freshwater resources [53].

Reports by Lee et al. point to the discovery of levansucrase isolated from *Sphingobium chunbukense* DJ77. The entire coding sequence of the *lsc* gene was cloned downstream of the T7 promoter and subsequently purified using the *Escherichia coli* protein expression system. The results suggest that recombinant *E. coli* carrying levansucrase from *S. chungbukense* DJ77 can produce levan under normal growth conditions with less need for pH manipulation [54].

*Gluconobacter japonicus* LMG 1417, due to its adaptability, genetic tractability and GRAS status, is a promising platform for the industrial production of levan [55]. LevS1417 levansucrase, produced by *G. japonicus* LMG 1417, and secreted by a signaling peptide-independent pathway, showed exceptionally high activity (4726 ± 821 U/mg at 50 °C). Cell-free levan production based on the supernatant of the tested strain led to a final levan concentration of 157.9 ± 7.6 g/L. The amount of levansucrase secreted was more than doubled by homologous plasmid-mediated overproduction of LevS1417 in *G. japonicus* LMG 1417. Therefore, the efficiency of cell-free levan production was doubled using a mutant containing a plasmid [55].

*Peanibacillus polymyxa* can be an extremely cost-effective source for the industrial production of levan exopolysaccharide (EPS) and its functional biomaterials for a wide range of applications, including bioengineering. Liyaskina et al. isolated *P. polymyxa* from honeycombs and sequenced its genome. Bioinformatic analysis identified the putative levan operon. The *sacC* and *sacB* genes were cloned and their products were identified as glycosidic hydrolase and levansucrase, respectively. The infrared spectra obtained through Fourier transform infrared spectroscopy (FT-IR) and the nuclear magnetic resonance (NMR) analysis revealed that the EPS (Exopolysaccharide) is composed of a linear fructan with β-(2→6) glycosidic bonds, specifically known as levan [56].

New endolevanase (*evB* 2286) from *Azotobacter chroococcum* DSM 2286, combines extremely high specific activity with beneficial hydrolytic properties. In contrast with endolevanases, LevB2286 produced small amounts of fructose and levan even with significantly prolonged incubation. The combined activity of LevB2286 and levansucrase LevS1417 from *Gluconobacter japonicus* LMG 1417 led to a one-step synthesis of levan fructooligosaccharide from sucrose [57].

Interestingly, in addition to bacteria, the yeast *Saccharomyces cerevisiae*, through heterologous expression of levansucrase, also can synthesize levan. Heterologous expression of levansucrase was very difficult to achieve in *S. cerevisiae*. As a strategy, Franken et al. used null mutant invertase (Δ*suc2*) and two separate, modified strains of yeast accumulating sucrose as hosts to express M1FT levansucrase, previously cloned from *Leuconostoc mesenteroides*. Intracellular accumulation of sucrose was achieved either by expressing sucrose synthase (Susy) from potato or spinach sucrose transporter (SUT). The data indicate that in both Δ*suc2* and sucrose-hoarding strains, M1FT was able to catalyze fructose polymerization. In the absence of the predicted M1FT secretion signal, intracellular levan accumulation was significantly increased for both strains of sucrose accumulation when they grew on minimal media [58]. 

Despite numerous years of research, levan synthesis and degradation continue to present intriguing avenues for investigation. The significance of the bimodal mass distribution of levan and the application of fractions with varying molecular weights remains unclear and requires further elucidation. The presence of branching within the levan molecule also poses many unresolved questions. It is yet to be determined whether the synthesis of branched polymers is genetically programmed within the microorganisms or if it is a consequence of enzyme activity and specific external conditions. There are indications that both the levansucrase and levanase operons mutually regulate each other, but the exact mechanisms are yet to be fully understood [47]. Additionally, it remains uncertain whether the presence of these operons in the genetic material of microorganisms guarantees their ability to synthesize levan, or if other factors are necessary for successful levan production. In many scientific articles, there is no exact characterization of the levan, and the structure and subsequent use may depend on the particular microorganism [37].

These challenges provide exciting directions for future research. Further investigations can focus on unraveling the molecular mechanisms underlying levan synthesis and degradation, including the regulatory interactions between relevant operons. Understanding the genetic control and environmental factors influencing levan branching and molecular weight distribution will contribute to a more comprehensive understanding of levan biosynthesis. Additionally, exploring the factors beyond operon presence that influences levan production will aid in optimizing levan synthesis strategies and potentially uncover novel pathways for enhanced levan production. Continued research in these areas will expand our knowledge of levan biology and pave the way for advancements in industrial applications and biotechnological utilization of this intriguing polysaccharide.

## 3. Microbial Preparation of Levan

Microbial levan is typically obtained through fermentation or enzymatic reactions using isolated enzymes. Sucrose serves as the substrate for the synthesis of this polysaccharide. The optimal sucrose concentration for achieving maximum synthesis efficiency varies not only among different species but also often among different strains of microorganisms, as evident from published data. Despite the various properties and potential uses of microbial polysaccharides, their industrial-scale utilization remains significantly lower compared to laboratory-scale applications. This is primarily due to the low efficiency of biomaterial synthesis, the multi-stage purification process and high production costs. Therefore, further research focusing on process optimization and product purification is crucial (Table 2). Furthermore, the fermentation duration undoubtedly affects the levan yield. Levansucrase, the enzyme involved in the synthesis, is hindered by the presence of residual post-reaction glucose. The accumulation of glucose in the production medium represents a bottleneck in large-scale levan production. Consequently, the microbiological production of this polymer remains limited at present. Commercially available levan is derived from *E. herbicola*, *Z. mobilis* or plants [59,60]. However, the limited quantities and high cost of commercial levan hinder its broader application, necessitating the ongoing search for alternatives in microbial production. An essential consideration in industrial levan production is the environmental impact associated with its manufacturing process. One stage of levan preparation involves precipitation using ethanol, methanol or acetone in varying proportions. The use of solvents and their subsequent recovery entail energy demands and have environmental implications. In certain cases, the pH of the solution is adjusted or other treatments are employed to cleanse it from the substrate before polymer precipitation [61,62,63,64]. Another strategy aimed at reducing costs and minimizing environmental impact involves the utilization of substitutes for raw materials required in polymer synthesis, often in the form of by-products derived from other industrial processes. The literature describes various approaches, including the use of sucrose substitutes like molasses or sugar syrups, alternative nitrogen sources or non-sterile production at high salt concentrations (Table 3).

**Table 2:** Obtaining levan from various microorganisms.

Scale	Microorganism	Sucrose Concentration [g/L]	Cultivation Conditions	Levan Concentration [g/L]	Literature
Laboratory	*Leuconostoc citreum* BD1707	172.0	112 h, 26 °C, 200 rpm, pH 6.12	34.86	[65]
	*Tanticharoenia sakaeratensis*	200.0	35 h, 37 °C, 250 rpm	24.70	[66]
	*B. subtilis* AF17	162.5	20 h, 30 °C, 150 rpm, pH 7.0	7.90	[67]
	*B.subtilis* MT453867	80.0	54 h, 37 °C, 150 rpm, pH 5.0	33.00	[68]
	*Brenneria goodwinii*	500.0	12 h, 35 °C, 8000 rpm, pH 6.0	185.00	[50]
	*Clostridium acetobutylicum*	28.00	72 h, 20 °C, - *, pH 6.0	- *	[51]
	*Leuconostoc mesenteroides*	90.0	24 h, 30 °C, - *, pH 5.0	- *	[52]
	*Sphingobium chungbukense*	10.00	24 h, 37 °C, - *, pH 5–10	- *	[54]
	*Gluconobacter japonicus* LMG 1417	720.00	24 h, 28 °C, 180 rpm, pH 6.8	157.90	[55]
	*Peanibacillus polymyxa*	124.00	96 h, 30 °C, 250 rpm, pH 7.2	68.00	[56]
	*Azotobacter chroococcum* DSM 2286 + *Gluconobacter* *japonicus* LMG 1417	809.6	48 h, 45 °C, - *, pH 6.2	387.40	[57]
	*Saccharomyces* *cereviasie*	50.00	48 h, 30 °C, pH 6.5	15.00	[58]
Bioreactor	*B. subtilis* M	100.0	150 L bioreactor, 24 h, 30 ℃, 200 rpm, pH 7.0, 0.5 vvm	47.00	[69]
	*B. subtlis* B58	130.0	16 L bioreactor, 16 h, 37 ℃, 400 rpm, pH 7.0, 1 vvm	26.65	[70]
	*Z. mobilis* B-14023	299.1	42.3 h, 28 ℃, pH 6.0	40.20	[59]
	*E. herbicola*	50.0	5 L bioreactor, 48 h, 25 ℃, 200 rpm, 0.2 vvm	4.50	[60]
	*B. subtilis AF17*	162.5	30 °C, 72 h, 150 rpm, pH 7.0	7.90	[67]
	*Halomonas smyrnensis* AAD6^T^	20.00	10 L bioreactor 37 °C, 200 rpm, 0.1 vvm,	18.06	[70]
	*Bacillus* *amyloliquefaciens*	20.00	5 L bioreactor, 37 °C, 48 h, 300 rpm, pH 6.0, 6 vvm	102.00	[71]
	*Pichia pastoris*	160.00	1,5 L bioreactor, 59 h, 28 °C, 200–1200 rpm	72.90	[72]
	*Saccharomyces* *cerevisiae*	191.00	50 L bioreactor, 48 h, 30 °C, 300 rpm, pH 5.5, 0.5 vvm	76.00	[73]

* not indicated

One of the primary by-products commonly utilized in levan research is molasses, which is derived from sugar production. Gojgic-Cvijovic et al. used diluted molasses in a medium and obtained 53.2 g/L levan during 48 h of fermentation of *B. licheniformis* NS032 [74]. In recent years, other studies have explored alternative uses of molasses as a sucrose source for levan production, employing strains such as *B. lentus* V8 [75] or *Brachybacterium phenoliresistens*, where molasses and date syrup have been used [76]. Another solution to the challenge of searching for substitutes for culture medium substrates is the process described by Erkorkmaz et al., in which the growth and yielding of levan by *Halomonas smyrnensis* was compared in a medium containing industrial sucrose from sugar beet syrup, salts of various origins as well as three different industrial boron compounds [77]. The most optimal culture medium, consisting of industrial food sucrose from sugar beets, sea salt from Çamaltı, industrial boric acid and borax pentahydrate, yielded 18.60 g/L levan, which is the highest yield recorded for *H. smyrnensis* to date [77].

**Table 3:** Examples of the use of various by-products in obtaining levan.

Substrate/By-Product used	Microorganism	Sucrose Concentration [%]	Levan Concentration [g/L]	Literature
Molasses	*Halomonas* sp. AAD6	48–51	12.40	[61]
	*B. licheniformis* NS032	49.40	53.20	[78]
	*B. lentus* V8	54	45.34	[75]
Sugar beet juice and syrup	*Z. mobilis* 113	65	9.60	[79]
The pulp after squeezing orange juice	*B. atrophaeus*	12–27	24.20	[63]
Distillers’ rye stock	*Z. mobilis*	*	25.17	[80]
Date syrup	*Microbacterium laevaniformans*	*	10.48	[81]
	*B. phenoliresistens*	*	8.12	[76]
Sugar cane syrup	*Z. mobilis*	*	15.46	[82]
Baklava syrup	*Z. mobilis*	*	8.90	[83]
Buckwheat sourdough bread	*G. albidus* TMW 2.1191; *Kozakia baliensis* NBRC 16680	8.86 ± 21.71; 50.26 ± 25.21	13.91; 12.65	[64]

* not indicated.

Levan is a multifunctional polymer of great industrial importance; hence, microorganisms characterized by its increased synthesis are still being sought. However, finding the right manufacturer is just the beginning of developing the process of receiving. Hence, it becomes important to improve strains using genetic engineering and optimization of the production process, to search for cheaper substitutes for expensive commercial microbiological substrates or to appropriate technology for obtaining the desired product for specific applications.

## 4. Properties and Application of Levan

Levan is a biodegradable, non-toxic and highly biocompatible compound, which makes it a very interesting polymer for the development of materials of natural origin. Levan exhibits a wide range of solubility, viscosity and stability depending on its source and production conditions. The properties depend on factors such as molecular weight concentration or branching and can range, for example, from highly soluble to partially soluble or insoluble.

Thanks to its properties, it is used in many fields, such as food production, modern cosmetology, medicine and pharmacy. The diversity in the structure of the levan molecule makes it a polymer that is still not fully understood.

**Figure 5:**
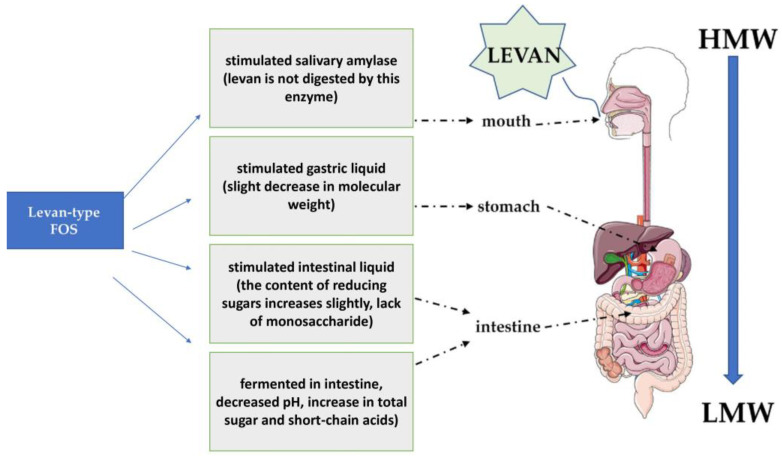
L-FOS and its specific prebiotic properties during digestion in humans based on [69].

In the food industry, levan is used as an emulsifying, stabilizing and thickening agent [46]. In addition, it can act as a sweetener and be a source of fructooligosaccharides with its prior hydrolysis [84,85]. Some levan fructooligosaccharides are more resistant to degradation in the gastrointestinal tract than their short-chain counterparts and have lower cholesterol levels [86,87]. Levan has prebiotic properties [88], promoting the growth of beneficial intestinal bacteria (Figure 5) [89]. It exhibits high thermal resistance, which makes it possible to process at high temperatures [90]. Recent research indicates the prebiotic properties of levan obtained via enzymatic synthesis. Xu et al. showed that levan increased the number of bacteria of the genus *Firmicutes*; mainly *Megasphaera* and *Megamonas* bacteria, and the proliferation of harmful genera was inhibited (*Cedecea* and *Klebsiella*) [91]. Levan also significantly increased total short-chain fatty acid levels, especially for propionic acid, butyric acid and valeric acid, which ensure the proper functioning of the intestines and accelerate the process of healing and regeneration of the intestinal epithelium. They also protect against pathogens (*Salmonella* spp., *Escherichia coli*, *Campylobacter* spp.) by lowering the pH of the environment in which they occur, inhibiting the multiplication of pathogenic microflora [92]. In their previous study, Xu et al. studied levan as a potential stabilizer of yoghurt. They proved that levan can increase the water-holding capacity (WHC) of fermented yogurt (over 77%) compared to fructoligosaccharides (FOS). In addition, levan showed better system stability (Zeta potential and WHC) than FOS. The addition of 0.2–0.5% obtained levan could increase the growth and sustainability of *L. bulgaricus* and *S. thermophilus* in fermented yoghurt [91].

Another interesting potential application of levan is the development of food packaging materials. Gan et al. tested its use in edible films with pullulan, chitosan and ε-polylysine that they coated strawberries with [93]. During their storage, they found that the weight loss of strawberries was lower and they retained their firmness and demonstrated antibacterial activity of films against *Escherichia coli* and *Staphylococcus aureus*, which are typical foodborne pathogens [93]. Koşarsoy-Ağçeli promotes an innovative approach to nanocomposite polymer-carbohydrate films using chia seed and levan mucus. In this case, for the first time, different degrees of mucus were obtained from chia seeds and the film-forming behavior of the levan biopolymer with these mucilages was studied. Films formed as nanocomposites of mucus, levan seeds and chia obtained at different temperatures exhibited different structural and mechanical properties. It was observed that films obtained with chia mucus and levan retained their antibacterial properties but lost their antifungal properties. These nanocomposites derived from chia seed mucilage and levan hold significant potential for applications in industrial and medical fields. Moreover, the environmentally friendly nature of these films plays a crucial role in promoting environmental protection [94].

Modern cosmetology continues to seek natural compounds that possess desirable properties in the beauty sector. In line with the current natural cosmetic trends, levan emerges as a suitable candidate due to its beneficial properties. Levan has been proven to be safe for human fibroblast cells and erythrocytes, making it a favorable ingredient in cosmetics [29,95,96]. Moreover, levan stimulates the proliferation of human keratinocytes and fibroblasts. It is non-irritating and can alleviate skin irritations caused by substances like sodium lauryl sulfate [97]. Levan, by inhibiting tyrosinase activity, and thus lowering melanin production, may act as a product regulating skin discoloration [98]. In vitro experiments by Kirtel et al. with microbiologically produced levan from *Halomonas* (HL) not only demonstrated increased proliferation of keratinocytes and fibroblasts, improved skin barrier function and increased type I collagen but also increased ability to rapidly heal wounds within 24 h on 2D wound models. This clearly shows that HL and its derivatives have a high potential for use as natural active ingredients in cosmeceutical and skin-regenerating preparations [53].

In the pharmaceutical industry, levan offers potential contributions to the stabilization and control of drug release in topical emulsion systems. Pantelić et al. have determined that levan is a compatible co-stabilizer with emulsifiers, cetearyl alcohol and sodium cetearyl sulfate, indicating its usefulness in preparations stabilized with anionic or non-ionic excipients [99]. Due to its ability to self-organize in water, it is an interesting component for the encapsulation of active compounds in the form of nanoparticles [100,101,102,103]. Furthermore, levan is also investigated for other “nano” forms, such as polymer micelles [104], nanocomposites [105] or nanoemulsions [103]. It could also be used to obtain hydrogels, e.g., for local skin antifungal therapy with amphotericin B [106]. Studies by Hamad et al. demonstrate the significant role of levan in the healing of burn wounds, accelerating them compared to base cream and saline [107]. Research by Osman et al. highlights the ease of dilution, washing, and loss of adhesion of levan in a humid environment. Researchers demonstrated a strategy for producing a levan-based self-adhesive hydrogel for hemostatic applications and wound healing by coupling catechol with levan. The prepared hydrogels showed significantly enhanced solubility in water and improved adhesion power to the hydrated skin of pigs. Hydrogels also promoted rapid blood clotting and much faster healing of rat skin incisions. Additionally, levan–catechol showed an immune response similar to a negative control response, attributed to significantly lower endotoxin levels compared to native levan [108].

It is worth mentioning that many studies indicate that the properties and subsequent use of levan are related to molecular weight and branching in the molecule. There are still few studies on the utilization of specific levan fractions [109,110] and they are often inconclusive. Previous studies have demonstrated that HMW levan exhibits anticancer activity [111,112] and lowers cholesterol [33], while the fraction of LMW levan has shown a better ability to encapsulate due to the possibility of slow and constant release of active substances resulting from its structure [102,113,114]. A more recent report shows that HMW levan can also be used to encapsulate xenobiotics [115]. Domżał-Kędzia et al. showed the cosmetic use of the LMW fraction of the biopolymer, indicating higher antioxidant properties than in the case of the HMW commercial levan from *E. herbicola* [29]. Charoenwongpaiboon et al. used ultrasound to reduce molecular weight and increase the bioavailability of levan. The bioactivity of both forms of levan (HMW and LMW) was tested using human osteosarcoma cells (*Saos-2*). The result clearly showed that the sonicated levan (LMW) had higher anti-proliferative activity in *Saos-2* cells than the original levan (HMW) [116]. Despite studies investigating the properties of various levan fractions, the mechanisms of action specific to each fraction and the relationship between structure and properties have not been fully elucidated. Further research in this area is warranted.

## 5. Levan Nanocarriers Development

**Figure 6:**
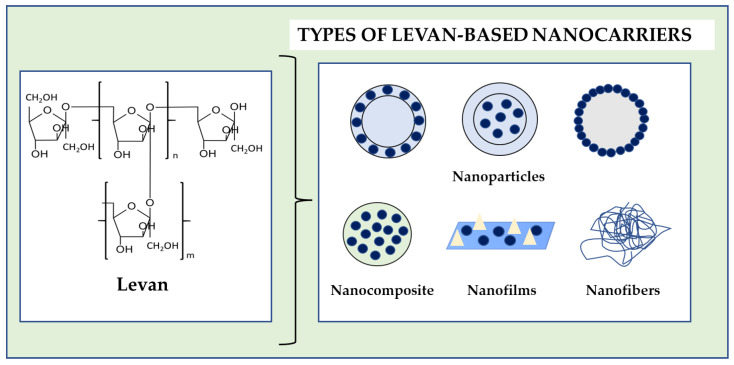
Examples of levan-based nanocarriers based on [90].

Levan is used in the study of various nanocarriers, such as nanofilms, nanocomposites, nano gels, nanofibers and nanoparticles (Figure 6) [110]. The direction of their applications is wide and includes issues related to food, nutrition, cosmetology, pharmacy and medicine. This shows how versatile the polymer is.

Axente et al. developed thin nanostructured layers of pure levan and its oxidized form using the matrix-assisted pulsed laser evaporation (MAPLE) technique. These layers can be a way of administering drugs and supporting tissue regeneration [117]. The obtained nanofilms were biocompatible with osteoblast cells and supported their adhesion, and the structure of oxidized levan increased cell proliferation, which is a process necessary for tissue regeneration. Bostan et al. obtained ternary films using chitosan, polyethylene oxide and levan by casting from a solution [118]. They have great potential to find applications as active food packaging and hemodialysis membranes and artificial leather materials. In addition, the presence of levan may support anticoagulant activity, soothing skin irritations, as a mixture component in cosmetics and supporting adhesion and cell proliferation.

Another application of levan lies in the field of nanocomposites, which are structures composed of at least two materials, with at least one of them being at the nanoscale [119]. An example of such a material is a composite of levan with montmorillonite clay, characterized enhanced thermal and rheological properties compared to pure levan [105]. Levan from *Paenibacillus polymyxa* was used to obtain a biodegradable film, also consisting of bentonite and selected essential oils with antimicrobial properties. The film containing peppermint oil showed the highest antimicrobial activity against *Candida albicans*, film containing lemon oil was effective against *S. aureus* and one with citronella showed activity against *E. coli* [120]. This solution has the potential to be used in biodegradable food packaging. Wang et al. developed a levan–chitosan (LE/CS) blend film by casting from a solution. The addition of levan significantly enhanced the absorption of UV light and reduced the swelling of water from the film layers [121]. LE/CS mixed films were used as packaging material for fresh pork and had well-maintained properties. The study suggests that the new blend film may have good prospects as a food packaging material. Song et al. developed a mechanically reinforced composite scaffold containing levan as a binder for sintered hydroxyapatite [122]. This scaffolding showed much greater mechanical strength, similar to bone cement made of polymethyl methacrylate. Their solution can be used in bone transplants and tissue engineering. Mujtaba et al. described the impact of composite coatings on the durability of cherry fruits. Composite coatings consisting of chitosan, chia mucus and levan were applied to the cherry fruit and tested for post-harvest parameters under both market and refrigeration conditions [123].

An example showcasing the utilization of levan properties in the preparation of hydrogels is the development of a topical injectable hydrogel, combining the biopolymer with Pluronic F127 and carboxymethylcellulose [124]. The resulting material was characterized by a higher modulus of elasticity, increased cell proliferation and higher expression of collagen synthesis in human skin fibroblast cells in in vitro tests compared to hydrogel based on hyaluronic acid. After injection during in vivo tests, it remained stable in the skin of mice longer than hydrogel from Pluronic F127 or with hyaluronic acid. Notably, the hydrogel demonstrated increased anti-wrinkle activity and stimulated collagen production in mice [125]. The subsequent combination of this hydrogel with hydroxyapatite resulted in prolonged anti-wrinkle activity and increased collagen production [126]. Demirci et al. used levan to develop a hydrogel with amphotericin B [106]. The distribution of this hydrogel was pH and temperature dependent, which allows for the local release of the drug. Levan itself stimulates skin cells to grow and supports regeneration, and additional substances incorporated into its structure allow for more therapeutic action in a precisely defined place. Additionally, modified levan proves to be a favorable material for hydrogel formation. Hydrogels based on hydrolyzed and phosphonate levan derivatives from *Halomonas* sp. cross-linked with 1,4-butanediol diglycidyl ether were characterized by a rigid gel structure with very good adhesive properties. Moreover, these hydrogels were loaded with resveratrol, and their biocompatibility was confirmed, opening possibilities for their application in tissue engineering and as local drug delivery systems [126].

A large group of levan-based carriers are nanoparticles. So far, several methods for obtaining levan nanoparticles have been described. These include nanoprecipitation [127], direct enzymatic synthesis [128], chemical reduction [129], self-organization [102], pyrolysis, co-precipitation [130], laser ablation [131] or electrohydrodynamic atomization techniques [101]. One of the greener processes for obtaining levan nanoparticles was proposed by Zhang et al., who obtained levan-based silver nanoparticles [132]. However, the process of obtaining them was multi-stage and required the use of organic solvents. Despite the ecological method of obtaining nanoparticles of levan with silver, the process of obtaining the polymer itself was not neutral to the environment and required the use of organic reagents other than ethanol and a large amount of energy. The vast majority of procedures for obtaining levan involve its precipitation with high-proof alcohol, and then the dried polymer is dissolved again to obtain nanoparticles. Scaling up production to produce equally effective nanoparticles requires a lot of energy and materials and is highly expensive.

Many scientists attempt to encapsulate various active compounds and drugs in the levan structure in the form of nanoparticles. Sezer et al. obtained microparticles of levan and vancomycin, showing that levan molecules could be a carrier for antibiotics and were safe [100]. Octavia et al. indicates that levan nanoparticles can encapsulate protein molecules and peptides, as demonstrated by albumin and lysozyme [133]. Cerium oxide nanoparticles, called nanocarriers, coated with levan showed antioxidant activity and reduced the level of ROS (reactive oxygen species) found after treatment of NIH/3T3 fibroblasts with hydrogen peroxide [130]. The research team led by Ko et al. developed a bifunctional protein carrier utilizing carboxymethyl levan for the cosmeceutical application of human epidermal growth factor (hEGF). The physiological effect of hEGF is often compromised due to its inherent low stability, particularly when exposed to the conditions of use. To address this issue, a stable delivery system utilizing levan-based nanoparticles has been developed. The utilization of carboxymethyl–levan significantly enhanced the entrapment efficiency of the various proteins examined. Moreover, the white carriers based on carboxymethyl–levan demonstrated remarkable functionality and stability [134].

Levan is also employed in the development of carriers specifically designed for cancer targeting. Kim et al. utilized self-assembly techniques to generate nanoparticles incorporating fluorescent indocyanine green [102]. In vivo imaging revealed the accumulation of these nanoparticles within breast cancer tumor. The presence of fructose in the levan structure enables binding to glucose transporter 5 (GLUT 5), which is overexpressed in breast cancer cells. Consequently, levan exhibits potential as a highly selective vehicle for targeted therapy [102,104]. In other cancer-focused studies, levan significantly improved the colloidal stability and encapsulation efficiency of doxorubicin in polymer-coated gold nanoparticles [131]. Furthermore, levan was used to coat lipid nanoparticles containing paclitaxel, resulting in controlled release of the drug over a period of six hours. These coated nanoparticles exhibited increased toxicity to human A549 lung cancer cells compared to uncoated nanoparticles, potentially due to the presence of levan in the coating [135]. Recent research on antitumor therapy reports the creation of nanoparticles with an intrinsic targeting ability to deliver anticancer drugs. Lee et al. addressed the short half-lives, lack of specificity and severe side effects of anticancer drugs by developing nanoparticles with active targeting capabilities. Levan has an affinity for binding to CD44 receptors and amphiphilicity. The nanoparticles self-assemble and allow active targeting without chemical modifications. Levan nanoparticles loaded with paclitaxel (PTX-LevNP) showed sustained PTX release and long-term stability. LevNP can bind CD44 receptors to cancer cells, and PTX-LevNP has shown increased antitumor activity in CD44-positive cells. In mice with SCC7 tumor, the accumulation of LevNP in the tumor tissue was 3.7 times higher than in the case of free dye, resulting in improved antitumor efficacy of the PTX-LevNP [136]. The process of obtaining levan had an impact on the physicochemical and biological properties of levan nanoparticles. Lewińska et al. studied two types of levan nanocarriers obtained from the *Bacillus subtilis* strain [137]. The first type of levan nanoparticles formed directly during the fermentation, while the second type was formed after levan was precipitated and redissolved in water. Notable differences in the surface morphology, free radical scavenging capacity and depth of penetration into the stratum corneum were observed between the two types of levan nanoparticles, indicating their potential applications in the cosmetics industry and pharmacy. So far, levan nanoparticles have been successfully implemented in cream, serum and tonic matrices, and their effectiveness has been confirmed in vivo [96]. 

## 6. Conclusions

Levan is still not fully understood as a polysaccharide. Levansucrase enzymes play a crucial role in levan biosynthesis, catalyzing the hydrolysis of sucrose and facilitating transfructosylation reactions. The regulation and interplay between levansucrase and levanase operons are still not fully understood. Various microorganisms have been investigated for levan production (also heterologous expression), including bacteria like *Bacillus*, *Zymomonas*, *and Halomonas* as well as baker’s yeast. The molecular weight, degree of branching and properties of levan can vary depending on the organism and reaction environment. The production of levan confers advantages to microorganisms in terms of energy storage, stress tolerance, biofilm formation and ecological adaptation, including symbiotic or pathogenic associations. Because biosynthesis occurs extracellularly; requires sucrose, which is not ubiquitously present outside living cells; and the reaction releases glucose into the environment, explaining the importance of levan production to various microorganisms will be challenging. Future research should focus on elucidating the molecular mechanisms underlying levan biosynthesis, exploring the regulatory interactions between operons, and optimizing levan production strategies. Understanding why levan microorganisms need it is not an easy task. Microorganisms live in very diverse environments and determining the importance of the levan for their existence seems crucial to the development of effective production. The precise determination of the mechanisms of formation of various fractions of levan was made possible by targeted actions within the genomes of microorganisms to improve the biosynthesis process and obtain a polymer with specific physicochemical and biological properties.

Microbial levan production involves fermentation or enzymatic reactions using isolated enzymes, utilizing sucrose as the substrate. The optimal sucrose concentration for efficient synthesis varies among different microorganism species and strains. Despite the wide range of potential applications of microbial levan, its industrial utilization remains limited due to low synthesis efficiency and high production costs. The presence of residual glucose inhibits levansucrase activity, posing challenges for large-scale production. Environmental considerations, such as solvent usage and recovery, pH adjustment and utilization of alternative raw materials, are important in industrial levan production. Molasses and other by-products have shown potential as substitutes for sucrose in levan synthesis. Continued efforts are needed to explore microbial strains with enhanced levan synthesis capabilities, genetic engineering for strain improvement, process optimization and the development of cost-effective production technologies to meet specific application requirements. For this purpose, it is worth paying attention to innovative technologies, both for obtaining and subsequent extraction and purification of levan. They are mainly based on the use of by-products from other industrial processes and can be based on a closed loop of the entire production cycle.

Levan demonstrates potential applications across various industries, as described in the scientific literature. Levan’s biodegradable and biocompatible nature, along with its diverse properties, make it an intriguing polymer for the development of a multitude of applications. It shows promise in the food industry as an emulsifying, stabilizing and thickening agent, as well as a sweetener and a source of fructooligosaccharides. Its prebiotic properties and ability to promote the growth of beneficial intestinal bacteria are noteworthy. Possible directions of research are the impact of levan impact on gut health, digestion, immune function and metabolic disorders. Understanding its role as a prebiotic and studying its potential applications in personalized nutrition and therapeutic interventions is another interesting direction of research. Levan also holds potential in food packaging materials, offering improved preservation and antimicrobial activity. In the food industry, it is necessary to investigate levan’s role in improving texture, flavor and nutritional value in food products. Developing innovative food formulations and processes that incorporate levan might meet consumer demands for healthier and functional foods. In the field of modern cosmetology, levan’s safety, moisturizing effects and regulation of skin discoloration make it an attractive ingredient. It has also been found to enhance skin barrier function, collagen production and wound healing. Furthermore, levan shows promise in pharmaceutical applications, such as stabilizing and controlling drug release, as well as in the encapsulation of active compounds.

In recent years, numerous carriers based on levan have been explored, demonstrating their considerable potential attributed to biocompatibility, biodegradability and non-toxicity. Extensive research has highlighted the significant possibilities of utilizing levan for health-promoting purposes. In particular, nano and micro-scale carriers have brought substantial advancements in the fields of healthcare, cosmetology and the food industry. Despite the long-standing knowledge of levan’s performance properties, its practical application has been limited in larger-scale settings due to inefficient production methods. Additional research is necessary to establish the effectiveness of levan-based pharmaceutical preparations on a larger scale. Currently, novel strategies are being introduced to improve the production and purification processes of levan. These efforts primarily aim to reduce costs and enhance the compound’s stability. Undoubtedly, further research, with a primary focus on isolating and purifying levan, will greatly contribute to its biotechnological applications.

## Figures and Tables

**Table 1:** Functions and applications of microbial polysaccharides.

Microbial Polymer	Synthesizing Microorganisms	Functions in Cell	Application in Industries	Literature
Alginate	*Azotobacter vinelandii*, *Pseudomonas aeruginosa*	Protection against desiccation, starvation, phagocytosis, UV radiation, effective barrier against reactive biocides, and involvement in the formation of a biofilm on a solid substrate. Under adverse conditions caused by changes in the ionic, osmotic, pH environment or toxic–metallic conditions, exopolysaccharides can create a protective buffer zone around the cell, protecting it from unfavorable changes in its structure. Increased ability of microorganisms to colonize host tissues.	For the production of dressings for hard-to-heal wounds, it is used for encapsulation, i.e., coating (immobilization) of various bioactive materials, as a component of face creams, In the food industry, this compound is used as a thickener, stabilizer or gelling agent in the production of jellies.	[16]
Bacterial cellulose	*Acetobacter* spp.		Cellulose dressings used to treat hard-to-heal wounds, e.g., burns.	[17]
Dextran	*L. mesenteroides*, *L. dextranicum*		In the food industry, it is used as a thickener and emulsifier, and in cosmetics, it is used as an ingredient in powders and lipsticks. However, the most important is dextran in medicine, where it can be used as a blood plasma substitute.	[18]
Gellan	*Sphingomonas elodea*		Stabilizing, thickening and gelling agent (E418). Due to its thermostability, gellan can be used as an alternative to microbial agar, in particular in the cultivation of thermophilic bacteria, and as a drug carrier.	[19]
Xanthan	*Xanthomonas campestris*		Stabilizing, thickening and gelling. Xanthan is used, e.g., for the production of puddings, sauces, drinks, fillings, and yoghurts, as well as in ointments, eye gels and toothpaste, and as an auxiliary substance in tablets, regulating the kinetics of the active substance release, e.g., by using the phenomenon of mucoadhesion—adhesion of the drug form to the membrane mucosa	[20,21]
Curdlan	*Alcaligenes faecalis*		Used primarily in the pharmaceutical and food industries, e.g., as a stabilizer, texture modifier and water binder.	[22]
Levan	*Bacillus* spp., *Lactobacillus* spp., *Pseudomonas* spp., *Streptococcus* spp., *Xanthomonas* spp., *Zymomonas* spp.		Stabilizer, flavor and fragrance carrier and thickener, and in the case of pharmaceuticals it can be used as a carrier of the active substance. In cosmetology, it is used for moisturizing preparations.	[23]
PHA	*Aeromonas hydrophila*, *Burkholderia sacchari*, *Escherichia coli*, *Halomonas baliviensis*		For the encapsulation of grains and fertilizers and the production of biodegradable containers and films, as sutures, orthopedic implants, mechanical barriers to prevent post-operative adhesions, internal drug release systems in the form of stents, scaffolding for bone marrow and bandages to support wound healing.	[24]
Pullulan	*Aureobasidium pullulans*		The production of edible films that are used in various breath fresheners or oral hygiene products, as a vegetarian substitute for medicine capsules, instead of gelatin, as a food additive is known under the number E 1204.	[25]

## Data Availability

Data are contained within the manuscript.

## Abbreviations

bp—base pair; BTE—Bone repair and Tissue Engineering; CMC—carboxymethyl cellulose; Da—Dalton; DLS—Dynamic Light Scattering; E1204—symbol of stabilizers, preservatives, thickeners; pullulan; E418—food additive symbol; gellan gum; EC 2.4.1.10—enzyme; levansucrase; EMD—Electrolytic Manganese Dioxide; EPS—exopolysaccharides; FDA—Food and Drug Administration; FOS—fructoligosaccharides; FT-IR—Fourier Transform Infrared Spectroscopy; GLUT 5—glucose transporter 5; GPC—Gel Permeation Chromatography (GPC); GRAS—Generally Recognized as Safe; HA—hyaluronic acid; HEGF—Human Epidermal Growth Factor; HL—levan from *Halomonas* spp.; HMW—High Molecular Weight; IgA—Imunoglobulins A; kDa—kilodalton; LE-CS—a levan-chitosan hybrid; L-FOS—levan type fructooligosaccharides; LMW—Low Molecular Weight; Lsc—levansucrase; M—mol; MAPLE—Matrix-Assisted Pulsed Laser Evaporation technique; NMR—Nuclear Magnetic Resonance; PCL—poly(ε-caprolactone); PF127—Pluronic F127; PHA—polyhydroxyalkanoates; PLA—poly(lactic acid); PTX-LevNP—levan nanoparticles loaded with paclitaxel; ROS—Reactive Oxygen Species; rpm—rotation per minute; Susy—sucrose synthase; SUT—sucrose transporter; vvm—air conditions used for bioreactor culture. The first ‘v’ stands for volume of air (e.g., liter); the second ‘v’ stands for per unit of medium (e.g., liter); ‘m’ stands for per unit of time (e.g., minute); WHC—Water Holding Capacity
